# First Report of Native Parasitoids of *Halyomorpha halys* (Hemiptera: Pentatomidae) in Greece

**DOI:** 10.3390/insects12110984

**Published:** 2021-10-31

**Authors:** Stefanos S. Andreadis, Nikoloz E. Gogolashvili, Georgios T. Fifis, Emmanouel I. Navrozidis, Thomas Thomidis

**Affiliations:** 1 Institute of Plant Breeding and Genetic Resources, Hellenic Agricultural Organization-Dimitra, P.O. Box 60458, 57001 Thermi, Greece; 2 Department of Agriculture, International Hellenic University—Sindos Campus, 57400 Sindos, Greece; gogolanick@gmail.com (N.E.G.); gakiasfif@gmail.com (G.T.F.); navrozidise@gmail.com (E.I.N.); 3 Department of Nutritional Sciences & Dietetics, International Hellenic University—Sindos Campus, 57400 Sindos, Greece; thomidis@ihu.gr

**Keywords:** *Halyomorpha halys*, brown marmorated stink bug, native egg parasitoids, *Anastatus bifasciatus*, *Ooencyrtus telenomicida*, biological control, Greece

## Abstract

**Simple Summary:**

The brown marmorated stink bug (BMSB) is a polyphagous species that causes severe damage to tree fruit, small fruit, vegetables, ornamental crops, and field crops. Classical biological control is one potential long-term and low-cost strategy to control the BMSB, using natural enemies. However, no natural enemy native to Greece that infects BMSB has been reported yet. Herein, we report the occurrence of two native hymenopteran egg parasitoids—*Anastatus bifasciatus* and *Ooencyrtus telenomicida*. Both egg parasitoids were collected from egg masses of the BMSB in the region of Thessaloniki, northern Greece. The total parasitism rate was 8.5%. Furthermore, *A. bifasciatus* was collected in mid-June and mid-August on egg masses of BMSB that were laid on green beans, apricots, and olives. On the other hand, *O. telenomicida* was only collected in mid-June, on one egg mass of BMSB that was laid on apricots. This first record could actually facilitate, for future tasks, the biological control of *H. halys* in Greece.

**Abstract:**

*Halyomorpha halys* (Stål) (Hemiptera: Pentatomidae) is an endemic species of East Asia; it was introduced into Europe in 2007. It has a wide range of hosts as it feeds on over 170 host plant species and significantly impacts crop production. In Greece, *H. halys* causes significant losses in the production of kiwi, peaches, and green beans; thus, control of this species (including biological control) is essential. Here, we focus on the potential impact of native natural enemies of *H. halys* in Greece. From June to October 2020, we sampled naturally field-laid *H. halys* egg masses to recover native parasitoids. A total of 20 egg masses of *H. halys* were collected from infested fields from different locations in northern Greece. Out of 529 eggs, 45 parasitoids managed to hatch successfully. The overall parasitism rate was 8.5%. We found two species of Hymenopteran egg parasitoids attacking *H. halys* eggs—*Anastatus bifasciatus* (Geoffrey) (Hymenoptera: Eupelmidae) and *Ooencyrtus telenomicida* (Vassiliev) (Hymenoptera: Encyrtidae), with the former comprising 58% of all parasitoids that were recovered. These results contribute to the knowledge about the natural enemy community that attacks *H. halys* in Greece, and the use of these native egg parasitoids in biological control programs may be a viable *H. halys* management strategy.

## 1. Introduction

The brown marmorated stink bug, *Halyomorpha halys* (Hemiptera: Pentatomidae), is a highly polyphagous pest that is native of Asia; it causes severe damage to a wide variety of fruit and vegetable crops by piercing the surface of the plant and fruit tissues [1,2].

*H. halys,* apart from being a major agricultural pest, is also considered a nuisance problem, because massive numbers of adults often invade in the fall and winter in residential and commercial buildings, to overwinter [3]. The earliest confirmed sighting in the United States was in 1996 in Allentown, Pennsylvania; since then, it has spread to 46 additional states and 4 Canadian provinces [3,4]. In Europe, *H. halys* was first discovered in 2007, in particular in Switzerland; within a few years, it managed to spread throughout all of Europe. Nowadays, it is considered a major threat to crops worldwide [5,6,7]. *Halyomorpha halys* was first mentioned in Greece in the fall of 2011, causing nuisance inside houses in several neighborhoods of Athens [8]. In 2017, in two different kiwi orchards in Northern Greece, located in the regional unit of Imathia and Pieria, respectively, significant damage on kiwi fruits was observed due to infestation by *H. halys*, and rendered them non-marketable [9]. In Greece, *H. halys* has two distinct generations [Andreadis, unpublished data].

Control of *H. halys* in the newly invaded areas, such as Greece, relies mainly on the use of broad-spectrum chemical insecticides [9]. However, chemical insecticides have met with mixed success in controlling *H. halys* due to the fact that they often lack efficacy [5,10,11], are harmful to the environment [12], incompatible with integrated pest management (IPM) strategies [10], and only provide a short-term solution. A potential long-term and low-cost strategy to reduce populations of the invasive agricultural pest *H. halys* is the classical biological control using natural enemies [13]. Nevertheless, no information is available regarding the potential for biological control through native parasitoids in Greece.

In its native habitat, *H. halys* is attacked by several natural enemies, including parasitoids [14,15,16] and predators [14,17], which target different development stages, causing significant mortality on its population [1]. Approximately 14 different parasitoid species of *H. halys* are known in its native range, of which, the most dominant are those that belong to the genera *Trissolcus*, *Telenomus* (Hymenoptera: Scelionidae), and *Anastatus* (Hymenoptera: Eupelmidae) [1]. For instance, in China, parasitism rates of *Trissolcus japonicus* (Ashmead) (synonymized by [18] with *T. halyomorphae* Yang) (Hymenoptera: Scelionidae) range up to 80% [16,17,19].

In North America, field surveys and studies that have been conducted in the past decades have led to the identification of several natural enemies of *H. halys*, mainly egg parasitoids; however, it was revealed that native egg parasitoids have not been effective at controlling newly laid *H. halys* eggs [6,20,21,22,23]. In fact, parasitism rates were typically low and highly variable among locations (and years) [5,6,24,25,26].

In Europe, several parasitoids attacking the eggs of *H. halys* have been reported [23,27,28,29], with *Anastatus bifasciatus* (Geoffroy) (Hymenoptera: Eupelmidae) being the most abundant native parasitoid among the few native egg parasitoid species; it is capable of developing on viable *H. halys* eggs [7,23,30,31,32]. Thus, it is as a potential candidate for augmentative biological control in Europe [30].

In Greece, regarding native natural enemies, neither parasitoids nor predators of *H. halys* have been reported. In the present study, carried out in Northern Greece, our main aim was to assess the occurrence of native parasitoids of *H. halys*, their parasitism rates, and relative abundance for potential application in biological control programs under local environmental conditions.

## 2. Materials and Methods

### 2.1. Description of the Study Area

**Figure 1:**
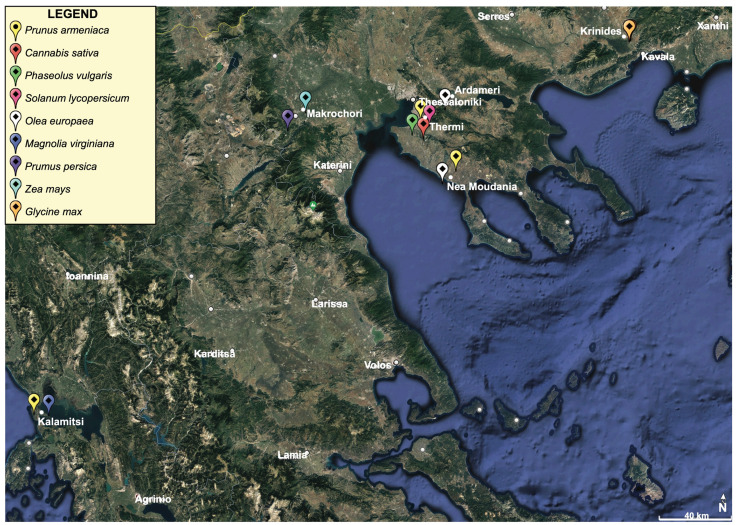
Satellite photo of the studied sites in Greece, coded by crop type (Google Earth © 2021 Maxar Technologies).

This study was conducted in the areas of Preveza (Kalamitsi), Chalkidiki (Nea Moudania), Veria (Makrochori), and Thessaloniki (Thermi, Ardameri) in Greece. Among the crops inspected were apricots (*Prunus armeniaca*) and magnolias (*Magnolia virginiana*) in Preveza; olives (*Olea europaea*) and apricots in Nea Moudania; peaches (*Prunus persica*) in Makrochori; apricots, industrial hemp (*Cannabis sativa*), tomatoes (*Solanum lycopersicum*), and beans (*Phaseolus vulgaris*) in Thermi; and olives in Ardameri (Figure 1). Apricots in Thermi and magnolias in Kalamitsi were mainly cultivated for family consumption or use, and no fertilizers or chemicals were applied at all. The surrounding environment was mainly dominated by pine trees. Tomatoes and beans in Thermi were located in small plots (less than 1 hectare) and grown next to other vegetables, such as peppers and cucumbers. The sizes of the rest fields ranged from 10 to 20 hectares and fertilizers and pesticides were used according to the farmers under different cultivation systems.

### 2.2. Collection of Egg Masses from the Field

A field collection of *H. halys* egg masses was carried out from June to October 2020 (Table 1). A total of 12 fields were inspected, including 5 fields in Thessaloniki, 2 fields in Chalkidiki, 2 fields in Imathia, 2 fields in Preveza, and 1 field in Kavala. Areas were selected based on previous reports of the presence of *H. halys*. As soon as the presence of *H. halys* in a field was confirmed, we started to inspect that field weekly. If the presence of *H. halys* was not confirmed for two consecutive weeks, then the inspection in the specific field stopped, resulting in unequal sampling per field. Both upper and lower surfaces of plant leaves were thoroughly inspected for egg masses. The number of plants that were randomly inspected varied among fields, depending on the severity of the infestation. When located, leaves with naturally laid egg masses were removed and placed individually in 50 mL falcon tubes. After collection, all egg masses were transferred to the Entomology Laboratory of the Institute of Plant Breeding and Genomic Resources (IPBGR), Thermi, Thessaloniki, Greece.

### 2.3. Laboratory Handling of Field-Collected Egg Masses

In the laboratory, *H. halys* eggs were counted separately by crop, region, and date of collection, and then individually transferred to clear plastic containers (460 mL), of glass vials (5 mL) filled with water. Egg masses, with the help of a fine brush (Artist’s Loft^TM^, MSPCI, Irving, TX, USA), were carefully placed on the upper surface of a green bean leaf from plants grown in the greenhouse at IPBGR, soaked in the glass vial. The containers where then covered with a plastic lid with mesh and placed in a room with a controlled environment at 26 °C, 65% RH, and a L16:D8 photoperiod. Egg masses were monitored daily and the number of parasitoids that hatched was recorded. Newly emerged parasitoids (<24 h) were placed in new clear plastic containers with a small cotton ball soaked in 10% honey–water solution, and a small plate with egg masses of *H. halys* from the laboratory rearing on the top of a filter paper. Emerged adult parasitoids from the laboratory-exposed egg masses of *H. halys* were preserved in small glass vials (5 mL), with 80% alcohol, and frozen at −20 °C. Vials with parasitoids were sent for morphological identification to the Natural History Museum of London (NHMUK). Voucher specimens were preserved at NHMUK.

## 3. Results

### 3.1. Distribution of Parasitoids

**Figure 2:**
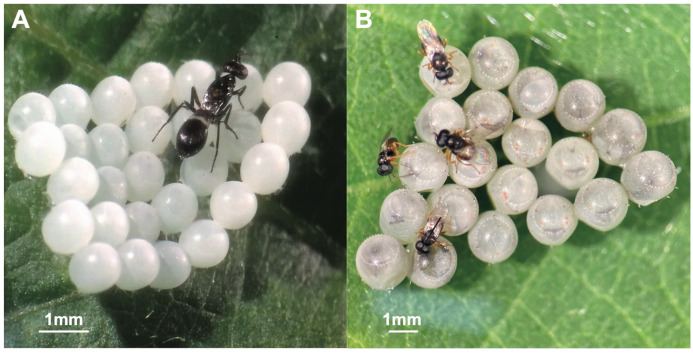
*Anastatus bifasciatus* (**A**) and *Ooencyrtus telenomicida* (**B**) observed on eggs of *Halyomorpha halys*.

Between June and October 2020, a total of 529 *H. halys* naturally laid eggs were sampled. However, only a small percentage of them were parasitized (8.5%). Two different parasitoids were identified: *Anastatus bifasciatus* (Geoffroy) (Hymenoptera: Eupelmidae) (Figure 2A) and *Ooencyrtus telenomicida* (Vassiliev) (Hymenoptera: Encyrtidae) (Figure 2B). Both parasitoids were sampled only in the regional unit of Thessaloniki. with *A. bifasciatus* present in two different locations, whereas *O. telenomicida* was only in the area of Thermi (Table 2).

### 3.2. Morphology of Parasitized Eggs

**Figure 3:**
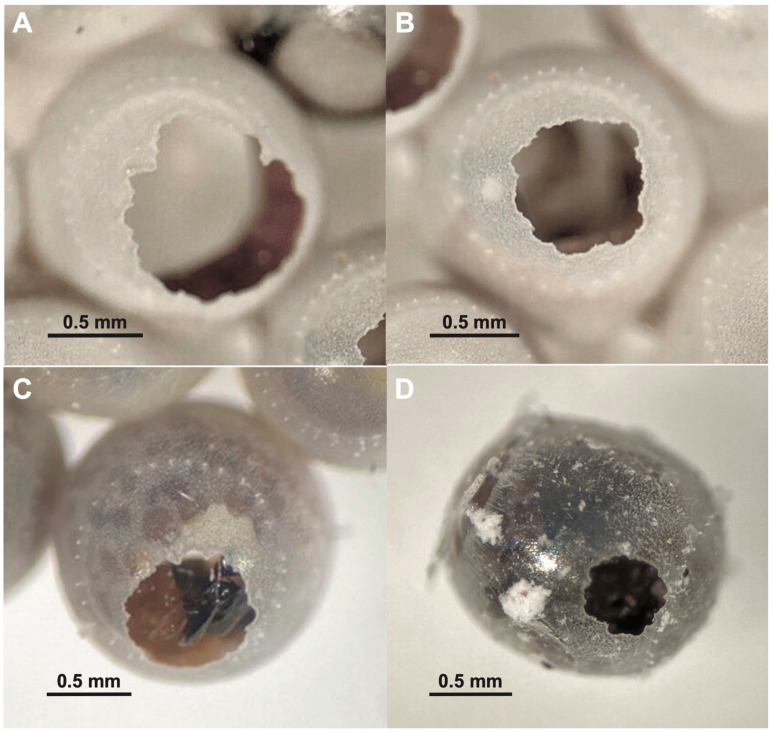
Typical shapes of exit holes produced by egg parasitoids of *Halyomorpha halys* in Greece: *Anastatus bifasciatus* female (**A**), and male (**B**), *Ooencyrtus telenomicida* female (**C**), and male (**D**).

Detailed observation of the parasitized eggs revealed distinct differences in the exit holes produced by *A. bifasciatus* and *O. telenomicida* in accordance with findings of a previous study by Sabbatini-Peverieri et al. [33]. Circular exit holes that are produced by males of *A. bifasciatus* (Figure 3A) are noticeably smaller than those of the larger females (Figure 3B). *Ooencyrtus telenomicida*, regardless of sex, produced exit holes in *H. halys* eggs similar in size or slightly smaller compared to that of male *A. bifasciatus*, but with much more irregular margins (Figure 3C,D).

**Figure 4:**
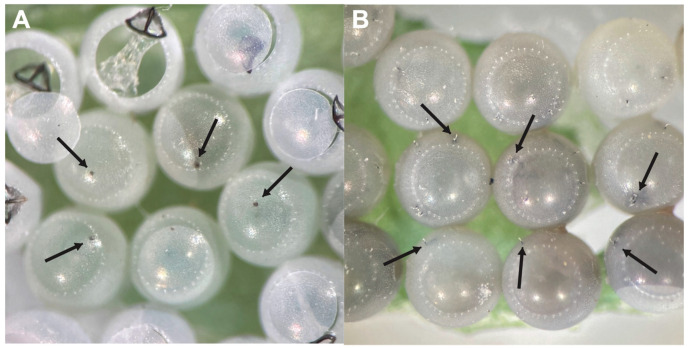
*Halyomorpha halys* eggs parasitized by *Anastatus bifasciatus* (**A**) and *Ooencyrtus telenomicida* (**B**). Arrows are pointing to oviposition sites of parasitoids.

Eggs of *H. halys,* in which we observed *A. bifasciatus* females insert their ovipositor, showed a characteristic dark spot on the egg chorion (Figure 4A). In the case of *O. telenomicida*, one or multiple egg stalks [34] protruding outside the host chorion were visible (Figure 4B).

## 4. Discussion

In our present field study, we observed two chalcid wasps, namely *A. bifasciatus* and *O. telenomicida*, to parasitize *H. halys* eggs. This is the first report of any natural enemy of the invasive species *H. halys* in Greece, exactly ten years since its first detection [8]. While *O. telenomicida* was observed only once in the season during mid-June, *A. bifasciatus* was observed thrice. It was first observed in mid-June in low numbers and again in the second half of August, in two different locations. This discovery adds important knowledge about the natural enemy community composition of *H. halys* in its native range, and could potentially be used for biological control in northern Greece.

In Europe, the generalist egg parasitoid *A. bifasciatus* has been reported on, with more than thirty-five hosts in the orders of Hemiptera, Lepidoptera, and Orthoptera [35,36]. It is among the most widespread native egg parasitoids of *H. halys,* and due to its ability to develop successfully on viable *H. halys* eggs, it can be considered a potential candidate for biological control [7,23,27,31,32,37,38]. Furthermore, *Anastatus* sp. is also part of the natural parasitoid community of *H. halys* in its native range in China [19]. In Greece, *A. bifasciatus* is a well-known egg parasitoid of the pine processionary caterpillar *Thaumetopoea pityocampa* (Denis & Schiffermueller) (Lepidoptera: Notodontidae) from the late 1980s [39]. Moreover, *A. bifasciatus* is part of a complex of primary egg parasitoids of *T. pityocampa*; however, infestation of *T. pityocampa* eggs by *A. bifasciatus* has been reported to be generally low [39,40,41,42,43]. Likewise, in most surveys, egg parasitism rates of *A. bifasciatus* on both naturally present and sentinel egg masses of *H. halys* were found to be less than 10% [27,37,44]. Its impact on *H. halys* is considered low, although it is frequently found throughout the survey region [32]. This is in accordance with our findings, as parasitism rates of *A. bifasciatus*, especially against naturally laid eggs of *H. halys,* in mid-June, was as low as 2.1%, while later, during the second half of August, it reached up to 8.0%. Interestingly, in our study, the emerged individuals of *A. bifasciatus* were of the female-based sex ratio (2.1:1), contrary to the ones that emerge from egg batches of *T. pityocampa,* where male individuals were the predominant or even the only ones [39,40,41,42,43]. This observation is of particular importance, contributing to the understanding of the sexual reproduction of *A. bifasciatus.*

The second egg parasitoid species that we detected was *O. telenomicida*, which is also considered a generalist egg parasitoid, attacking mainly heteropteran as well as lepidopteran species [45,46,47]. *Ooencyrtus telenomicida* is a dominant species in the Mediterranean climatic regions whose abundance is positively correlated with annual rainfall [48]. Recently, Roversi et al. [23] reported *O. telenomicida* from sentinel eggs of *H. halys* from a non-infested area in Tuscany, Italy. However, although *O. telenomicida* is able to complete development to the adult stage within its host *H. halys*, such as *A. bifasciatus*, abundance of this species in the field is generally low. In Western Slovenia, only one egg mass that was exposed in an agricultural area was parasitized by *O. telenomicida* [49], while in another study, in the region of Emilia-Romagna, Italy, none of the 11,841 sentinel eggs that were exposed were ever parasitized by this species [32]. This may be explained by the climatic and environmental differences between the surveyed regions or the local availability and abundance of the natural host(s) of *O. telenomicida* [32]. As in the case of *A. bifasciatus*, the sex ratio of *O. telenomicida* was female biased (1.86:1), in accordance with that recorded in a laboratory study in Italy, when *O. telenomicida* was reared on *Graphosoma lineatum* (Hemiptera: Petatomidae) eggs at temperatures above 26 and 30 °C [50]. According to the literature available, the host species does not affect sex ratio in *O. telenomicida* [47,48]; however, temperature does play a role in influencing sex ratio with a decrease in the number of females associated with lower temperatures [50]. Interestingly, *O. telenomicida* seems to be more frequent in mid-June, while *A. bifasciatus* appears to be in higher numbers during the second half of August, which is in contrast to the findings of a previous study conducted in Central Italy [23].

During the present study, a total of nine *H. halys* hosts were confirmed (Table 1). *Prunus armeniaca*, *Olea europea*, *Cannabis sativa*, and *Phaseolus vulgaris* were the most common hosts in the agricultural area. Apricot was the main oviposition host from early June to late July, with seven egg masses collected, almost two-thirds of all *H. halys* egg masses collected during that period. On the other hand, green beans were the main hosts from early August to early October, with eight egg masses collected during that period. Moreover, *H. halys* oviposited, based on the relative abundance of potential host plants. Both apricot and green beans are reported among the most acceptable hosts of *H. halys* in the invaded areas, suitable for oviposition and nymphal development [6,51]. *Anastatus bifasciatus* was collected in mid-June and mid-August on egg masses of *H. halys* that were laid on green beans, apricots, and olives. On the other hand, *O. telenomicida* was collected only on mid-June on one egg mass of *H. halys* that was laid on apricots. Although both species are considered as generalist egg parasitoids, *A. bifasciatus* is likely more widespread and present in more habitats compared to *O. telenomicida*.

The search for native natural enemies as an alternative solution against invasive pests in field crops has been considered recently, particularly for *H. halys* [7,23]. Their success strongly depends on a thorough understanding of the biology and ecology of both the pest and natural enemy [2,52,53]. Thus, the identification of native natural enemies and egg parasitoids, particularly associated with *H. halys,* and the evaluation of their impact, is needed, prior to any biological control implementation.

## 5. Conclusions

The present study provides basic information on the presence and species diversity of egg parasitoids that are associated with *H. halys* in Northern Greece. More specifically, the data obtained during this study provide a solid basis for future tasks regarding the biological control of *H. halys* in Greece. Hence, a better understanding of the specific roles of *A. bifasciatus* and *O. telenomicida* as biological control agents of *H. halys* in field crops, as well as of the interactions between these two species, is essential for providing further tools, in terms of a robust and sustainable IPM strategy.

## Figures and Tables

**Table 1:** Data on field survey from June to October, 2020, in Greece.

No.	Region	Locality	Location Type	Coordinates (Latitude, Longitude)	Host Plant
1	Thessaloniki	Thermi	Suburban area (fruit orchard, ornamental trees)	40°32′17″ N 23°00′04″ E	*Prunus armeniaca*
2	Thessaloniki	Thermi	Agricultural area (field crops)	40°32′06″ N 23°00′22″ E	*Cannabis sativa*
3	Thessaloniki	Thermi	Suburban area/Agricultural area (field crops, vegetables)	40°32′12″ N 23°00′02″ E	*Phaseolus vulgaris*
4	Thessaloniki	Thermi	Suburban area/Agricultural area (field crops, vegetables)	40°32′13″ N 23°00′01″ E	*Solanum lycopersicum*
5	Thessaloniki	Ardameri	Agricultural area (extensive olive orchards)	40°36′00″ N 23°09′10″ E	*Olea europaea*
6	Chalkidiki	Nea Moudania	Agricultural area (intensive fruit orchards)	40°15′43″ N 23°16′17″ E	*Prunus armeniaca*
7	Chalkidiki	Nea Moudania	Agricultural area (intensive olive orchards)	40°15′23″ N 23°16′23″ E	*Olea europaea*
8	Preveza	Kalamitsi	Suburban area (ornamental trees)	38°58′18″ N 20°43′16″ E	*Magnolia virginiana*
9	Preveza	Kalamitsi	Suburban area/Agricultural area (extensive fruit orchard)	38°58′17″ N 20°43′08″ E	*Prunus armeniaca*
10	Imathia	Makrochori	Agricultural area (intensive fruit orchards)	40°33′34″ N 22°17′16″ E	*Prunus persica*
11	Imathia	Makrochori	Agricultural area (field crops)	40°33′37″ N 22°17′19″ E	*Zea mays*
12	Kavala	Krinides	Agricultural area (intensive field crops)	40°59′51″ N 24°19′40″ E	*Glycine max*

**Table 2:** Data on naturally laid egg masses of *Halyomorpha halys* collected from June to October 2020 from different locations in Greece.

Region	Locality	Host Plant	Collection Date	No. of Egg Masses	No. of Eggs	No. of Parasitoids Emerged
Thessaloniki	Thermi	*Prunus armeniaca*	8/6	2	56	
			11/6	2	55	
			14/6	1	14	6 AB (4♀, 2♂)
			20/6	2	55	20 OT (13♀, 7♂)
			27/6	0	-	-
			4/7	0	-	-
		*Cannabis sativa*	17/6	1	27	0
			20/6	1	28	0
			27/6	0	-	-
			4/7	0	-	-
		*Phaseolus vulgaris*	20/8	2	56	16 AB (11♀, 5♂)
			28/8	2	56	
			3/9	0	-	-
			9/9	2	56	0
			14/9	2	56	0
			21/9	0	-	-
			28/9	0	-	-
		*Solanum lycopersicum*	29/9	0	-	-
			5/10	0	-	-
			12/10	0	-	-
	Ardameri	*Olea europaea*	19/8	1	14	3 AB (2♀, 1♂)
			26/8	0	-	-
			2/9	0	-	-
Chalkidiki	Nea Moudania	*Prunus armeniaca*	4/6	0	-	-
			11/6	0	-	-
			18/6	0	-	-
		*Olea europaea*	11/6	0	-	
			18/6	0	-	-
			25/6	0	-	-
Preveza	Kalamitsi	*Magnolia virginiana*	25/6	2	56	0
			2/7	0	-	-
			9/7	0	-	-
		*Prunus armeniaca*	3/6	0	-	-
			10/6	0	-	-
			17/6	0	-	-
Imathia	Makrochori	*Prunus persica*	14/7	0	-	-
			21/7	0	-	-
			28/7	0	-	-
		*Zea mays*	14/7	0	-	-
			21/7	0	-	-
			28/7	0	-	-
Kavala	Krinides	*Glycine max*	25/8	0	-	-
			1/9	0	-	-
			8/9	0	-	-
			Total	20	529	45

AB = *Anastatus bifasciatus*, OT = *Ooencyrtus telenomicida*.

## Data Availability

The data presented in this study are available on request from the corresponding author.
